# Fish oil supplementation reverses the effect of cholesterol on apoptotic gene expression in smooth muscle cells

**DOI:** 10.1186/1476-511X-9-70

**Published:** 2010-07-14

**Authors:** Sonia Perales, Ma José Alejandre, Rogelio Palomino Morales, Carolina Torres, Ana Linares

**Affiliations:** 1 Department of Biochemistry and Molecular Biology I, Faculty of Sciences, Campus Universitario de Fuentenueva Avenida Severo Ochoa s/n 18071 University of Granada, Spain

## Abstract

**Background:**

Nutritional control of gene regulation guides the transformation of smooth muscle cells (SMC) into foam cells in atherosclerosis. Oxidative stress has been reported in areas of lipid accumulation, activating proliferation genes. Suppression of oxidative stress by antioxidant administration reduces this activation and the progression of lesions. We hypothesized that fish oil consumption may protect against atherosclerotic vascular disease. The study objective was to determine the effects of dietary cholesterol and fish-oil intake on the apoptotic pathways induced by 25-hydroxycholesterol (25-HC) in SMC cultures.

**Methods:**

An *in vivo/in vitro *cell model was used, culturing SMC isolated from chicks exposed to an atherogenic cholesterol-rich diet with 5% of cholesterol (SMC-Ch) alone or followed by an anti-atherogenic fish oil-rich diet with 10% of menhaden oil (SMC-Ch-FO) and from chicks on standard diet (SMC-C). Cells were exposed to 25-HC, studying apoptosis levels by flow cytometry (Annexin V) and expressions of caspase-3, c-myc, and p53 genes by quantitative real-time reverse transcriptase-polymerase chain reaction. Results: Exposure to 25-HC produced apoptosis in all three SMC cultures, which was mediated by increases in caspase-3, c-myc, and p53 gene expression. Changes were more marked in SMC-Ch than in SMC-C, indicating that dietary cholesterol makes SMC more susceptible to 25-HC-mediated apoptosis. Expression of p53 gene was elevated in SMC-Ch-FO. This supports the proposition that endogenous levels of p53 protect SMC against apoptosis and possibly against the development of atherosclerosis. Fish oil attenuated the increase in c-myc levels observed in SMC-C and SMC-Ch, possibly through its influence on the expression of antioxidant genes.

**Conclusion:**

Replacement of a cholesterol-rich diet with a fish oil-rich diet produces some reversal of the cholesterol-induced changes, increasing the resistance of SMC to apoptosis.

## Background

Gene expression is influenced by dietary fat [1], gene transcription is regulated by dietary polyunsaturated fatty acids [2], and the nutritional control of gene regulation guides the transformation of SMC into foam cells in atherosclerosis [3]. The present study focused on the effect of cholesterol and fish oil intake on the expression of apoptosis-regulating caspase-3, c-myc and p53 genes.

In the 1970 s, a very low incidence of death from ischemic heart disease was observed in Greenland Eskimos, who have a high fish consumption [4]. Evidence from several research lines supports the hypothesis that fish consumption may protect against atherosclerotic vascular disease. Fish oil inhibits development of atherosclerosis in rhesus monkeys, rabbits, and humans [5-7], the n-3 fatty acids present in fish oil also have several biological effects that may be antiatherogenic. In the last decade evidence had been provided, that other fatty acids compounds are responsible for the positive effects of fish oil, e.g. furan FA (F-acids). F-acids are generated in large amounts in algae, but they are also produced by plants and microorganisms. Fish and other marine organisms as well as mammals consume F-acids in their food and incorporate them into phospholipids and cholesterol esters. The biogenetic precursor of the most abundant F-acid, F6, is linoleic acid. F-acids react readily with peroxyl radicals to generate dioxoenes. The radical-scavenging ability of F-acids may contribute to the protective properties of fish and fish oil diets against mortality from heart disease. [8]. However, the mechanism by which fish oil exert their effects is not fully understood. It has been proposed that some of these effects occur *via *plasma lipids [9]. It is well established that menhaden oil supplementation of the diet of chicks produced drastic reductions in plasma cholesterol, VLDL, and low- and high-density lipoprotein (LDL and HDL) fractions [9]. Supplementation with 10% menhaden oil was also reported [10] to drastically inhibit of 3-hydroxy-3-methylglutaryl-CoA (HMG_CoA) reductase activity in neonatal chick liver. In another study, supplementation of neonate chick diet with 10% menhaden oil for 7 days resulted in a significant hypocholesterolemia and hypotriglyceridemia [11].

However, the effects of fish oil on the regulation of apoptosis are less well known [6]. It has been reported that free cholesterol loading of macrophages induces apoptosis in atherosclerosis [12,13] and that apoptosis decreases after lipid lowering [14]. Furthermore, smooth muscle cells (SMC) in atherosclerotic plaques can be lost *via *apoptosis [15], destabilizing plaque and increasing the risk of thrombosis. The decision phase of apoptosis involves the expression of specific pro- and anti-apoptotic genes. If the sum of signals gives apoptosis as a result, the entire protein machinery for destruction is unleashed. In SMC, apoptosis can be promoted by deregulation of the expression of c-myc or E2F [16], which in turn can be regulated by p53. The expression of p53 alone can promote apoptosis in SMC [17]. In atherosclerosis, SMC are highly susceptible to p53-mediated apoptosis [18].

25-hydroxycholesterol (25-HC) induced apoptosis in a leukemia model system [19], and 25-HC treatment repressed expression of the proto-oncogene c-myc, an early response gene [20,21] whose product, c-Myc, is critical for the control of cell proliferation and differentiation [22]. It has also been reported that oxLDL and α-tocopherol may influence c-Myc activation in human coronary SMC [23]. Enhanced expression of c-Myc mRNA was observed in human SMC cultured from aortic plaques [24], carotid atherosclerotic lesions [25], and vein graft hyperplasia [26]. The tumor suppressor gene p53 encodes a transcription factor that activates genes involved in growth arrest (p21, GADD45) and apoptosis (Bax, Fas, p53-induced genes) [27]. p53 induces apoptosis by transcriptional activation of proapoptotic genes/repression of anti-apoptotic genes and also *via *non-transcriptional mechanisms. The cellular response to p53 depends on the cell type and p53 expression level and on the presence of other apoptotic stimuli. Thus, low-level p53 expression often induces growth arrest, whereas apoptosis is only induced by higher expression levels. The induction of growth arrest may also reduce the sensitivity of cells to apoptosis [27]. In human atherosclerosis, p53 expression is negatively correlated with cell proliferation markers [28]. Endogenous p53 levels protect vascular SMC and stromal cells against apoptosis while promoting apoptosis in macrophages, and they protect against atherosclerosis development [29]. It was reported that p53 is highly expressed in atherosclerotic lesions and is involved in oxidized LDL-induced apoptosis [30].

Our group has developed an experimental culture model of SMC isolated from aorta of cholesterol-fed chicks (Ch-SMC) and control chicks on a standard diet (C-SMC) cultured under the same conditions. It has proven suitable for *in vitro *study of the transformation into foam cells of SMC induced *in vivo *by cholesterol diet, isolating the cells before plaque formation [31]. Previous results with this model showed marked differences in proliferation and synthesis of DNA, RNA and protein between Ch-SMC and C-SMC, and these cell lines differed in lipid (especially cholesterol) synthesis and in the effects of inhibition on the first step of cholesterol biosynthesis [31,32]. In another study, Ch-SMC and C-SMC cultures showed altered HMG-CoA reductase activity and gene expression at transcriptional level [33]. Hence, a high-cholesterol diet induced changes in the HMG-CoA reductase gene expression in aortic SMC of the chicks. The HMG-CoA reductase mRNA concentration in SMC cultures showed a marked rise after feeding that was not correlated with the fluctuation of activity observed during feeding of the cells for 72 h [34].

The hypothesis of this study was that vascular SMC apoptosis gene expression may be affected during early atherosclerosis in the *in vivo/in vitro *SMC culture model of chick experimental hypercholesterolemia and that fish oil supplementation after cholesterol dietary could change these results. We previously characterized 25OHC-induced SMC apoptosis by studying antiapoptotic and proapoptotic (bcl-2, bcl-xl and bax) gene expression [35]. The objective of the present study was to characterize 25-HC-induced SMC apoptosis by studying of caspase-3, c-myc, and p53 gene expression in SMC-C, SMC-Ch, and SMC-Ch-FO cultures. SMC-Ch-FO cultures were isolated from aorta of chicks fed with high-cholesterol diet for 10 days and then with standard diet supplemented with 10% fish oil for a further 10 days. The results showed that the effects of the cholesterol diet on the expression of these genes were reversed by dietary supplementation with fish oil.

## Methods

### Animals

The protocol of this study was approved by the Animal Laboratory Service of the University of Granada (Spain), and the chicks received humane treatment according to the regulations for Animal Research of the European Union. Newborn White Leghorn male chicks (Gallus domesticus), supplied by the Animal Laboratory Service of the University of Granada, were kept in a chamber with a light cycle from 9 am to 9 pm and controlled temperature of 29-31°C with food and water available *ad libitum*.

### Diet and treatment

The diet was started at hatching and maintained until the chicks were killed at 20 days. Water was always available. None of the chicks died a natural death during the treatment or developed any disease. Three groups of 20-day-old chicks were used: control diet group (C-group), kept on a standard diet (Sanders A-00); Ch-treatment group (Ch-group), fed on the same diet supplemented with 5% w/w powdered cholesterol mixed homogeneously (Panreac reagent Barcelona, pure grade); and a Ch-fish oil group (Ch-FO-group), fed for 10 days with the 5% cholesterol-supplemented diet and then for a further 10 days with the standard diet supplemented with 10% fish (menhaden) oil. Experimental diets were prepared daily to minimize oxidation. The standard diet contained (w/w) 42% carbohydrate (mainly starch), 3.5% fat and 20.5% protein. The fatty acid composition of the diets is given in Table 1[11]. No significant differences in fatty acid composition were found among the diets. After the treatments, animals were anaesthetized with ketamine (60 mg/kg of body weight) and sodium pentobarbital (50 mg/kg of body weight) before decapitation and removal of the aortic arch. No animals suffered at any stage of the experiment, and the study protocol was approved by the ethics committee of our university.

**Table 1:** Fatty acid composition of control and experimental diet.

Fatty acid	Control	+ 10% fish Oil
14	0.8	6.6
16	22.3	22.3
18	8.6	5.8
Total sat.	31.7	34.8
		
16:1 n-7	3.3	9.2
18:1 n-9	32.4	19.5
20:1 n-9	0.0	0.4
Total MUFA	35.7	29.1
		
18:2 n-6	24.6	10.7
20:2 n-6	2.5	2.4
20:3 n-6	1.1	0.6
20:4 n-6	1.6	1.4
Total n-6	29.8	15.1
		
18:3 n-3	0.8	0.6
20:5 n-3	0.0	12.0
22:5 n-3	1.7	1.6
22:6 n-3	0.0	6.8
Total n-3	2.5	21.0
		
Total PUFA	32.3	36.1
Total unsat.	68.0	65.2
Sat./unsat.	0.47	0.53
Sat./PUFA	0.98	0.96
		
20:5/22:6	0.00	1.76
n-3/n-6	0.08	1.39

Source: Castillo *et all *[11]

Results (% fatty acid) are the means of 3 determinations.

### Smooth muscle cell culture

SMC were isolated from the aortic arch of the chicks as described elsewhere with slight modifications [31] and cultured in Dulbecco's Modification of Eagle's Medium (DMEM) supplemented with D-glucose (4.5 g/l), L-glutamate (0.584 g/ml), antibiotic cocktail composed of penicillin (100 mg/ml) and amphotericin (0.25 mg/ml) (Sigma-Aldrich, Inc), and 10% (v/v) fetal bovine serum (FBS). Medium was buffered with bicarbonate, and cultures were kept at 37°C in humidified atmosphere (95% air, 5% CO_2_). Media were renewed three times a week. Secondary cultures were initiated after either low or high passages using 0.05%/0.02% Trypsin-EDTA solution. All experiments were conducted using 3 or 5 passages. Cells were identified as vascular SMC by their hill-and-valley configuration at confluence and positive fluorescence staining for smooth muscle actin and myosin.

### 25-hydroxycholesterol treatment and cytotoxicity assay

Cytotoxicity was analyzed by using the MTT assay (3-(4,5-dimethylthiazol-2-yl)-2,5-diphenyltetrazolium, bromide; Sigma). SMC were plated in 96-well plates at a density of 25 000 cells/well. After adhering during overnight culture, cells were treated for 24 and 48 h with 25-HC (5-40 μg/ml) dissolved in absolute ethanol. The final concentration of ethanol in the culture medium never exceeded 0.8% and no effect on culture was observed at or below this concentration.

MTT was dissolved in DMEM at a concentration of 5 mg/ml. An amount of this solution equal to 10% of the culture medium volume was added to cell cultures. After 2 h, cultures were removed from the incubator and washed with PBS. The formazan crystals were solubilized by adding 200 μl of solubilization solution (0.05 N HCl in isopropanol). Metabolic activity was quantified by subtracting light absorbance at 630 nm from absorbance at 570 nm.

### Apoptosis Assays by Flow Cytometry

Apoptotic cell death in SMC was assessed by flow cytometry (Annexin V-FLUOS Staining Kit, Roche). The culture medium of each plate (containing cells detached during the cell death process) was recovered in a tube, and the cells were suspended by a brief trypsinization (0.05% with EDTA) and washed twice with cold PBS. Cells were then resuspended in 100 μL of 1x binding buffer (10 mM Hepes, pH 7.4, 150 mM NaCl, 2.5 CaCl_2_, 1 mM MgCl_2_, 4%BSA) to a density of 1 × 10^6 ^cells/ml with annexin V-fluorescein isothiocyanate (FITC) (0.5 μg/ml). Cells were gently mixed and incubated for 15 min at room temperature in the dark. They were then re-suspended in 1x binding buffer, and propidium iodide (PI) (0.6 μg/mL) at 4°C was added to each tube before analysis. Fluorescence was induced with the 488-nm argon laser and monitored at 512 nm (FL1) for the FITC signal and 620 nm (FL2) for IP fluorescence on FACS Vantage (Becton Dickinson Immunocytometry System, San José, CA.). The log of annexin V-FITC fluorescence was plotted against the log of IP fluorescence.

### Real-Time PCR Analysis

Total RNA was isolated with Tri-Reagent/Trizol (Invitrogen, Ltd, UK). Single-stranded cDNA was synthesized from 4 μg total RNA using an Oligo(dT)_12-18 _as primer and PowerScript™ reverse transcriptase (Clontech Laboratories, Inc., CAL). Real-time PCR was performed with the Fast Start DNA Master SYBR Green I Kit (Roche) and Light Cycler system (Roche). For the Light Cycler reaction, a master mix of the following reaction components was prepared to the indicated final concentration: 12.6 μL H_2_O, 2.4 μl MgCl_2 _(4 mM), 1 μL forward primer (0.5 μM), 1 μL reverse primer (0.5 μM), and 2.0 μL of the Fast Start DNA Master SYBR Green I mix (Roche). The primer sequences used in this study are listed in Table 2 and were optimized at an annealing temperature of 55°C. The cDNA of the genes studied in the different samples (treated *in vivo *and *in vitro*) were diluted 1:100 and amplified to obtain the Cp value for each sample. Light Cycler products of the different gene expressions were analyzed by agarose gel electrophoresis, and a Light Cycler melting curve was constructed to test for a single product at the end of each PCR reaction. A mathematical model developed by Pfaffl [36] was used for the relative quantification of caspase 3, c-myc, and p53 mRNA expression in real-time PCR with respect to the reference β-actin gene transcript.

**Table 2:** Primer sequences and PCR product lengths for β-actin, caspase 3 c-myc and p53.

Gene	Forward primer	Reverse primer	length
α-actin αβ-actin	GCTCCGGCAATGTGCAA	AGGTTCATGAGGTAGT	515
c-myc	AGCGAACGAGTCTGAATCCAGC	TTCAACTGTTCTCTCTCCTCCGCC	476
p53	ATGTGCAACAGTTCCTGCAT	AGTTCTCCTCCTCGATCTTG	160
caspase 3 3-3	TTAGATTCTGGTATTGAAGC	GAAATCCTGTCGAGTGGAGCAGG	269

### Statistical analysis

Results are expressed as mean ± SEM. Data were analyzed by Student's *t*-test or, when the variances of two datasets significantly differed, by Welch's alternate *t-*test InStat statistical package was used for the data analyses. A *p*-value ≤ 0.05 was considered significant.

## Results

### 25-hydroxycholesterol induces apoptosis in the three cell cultures types

**Figure 1:**
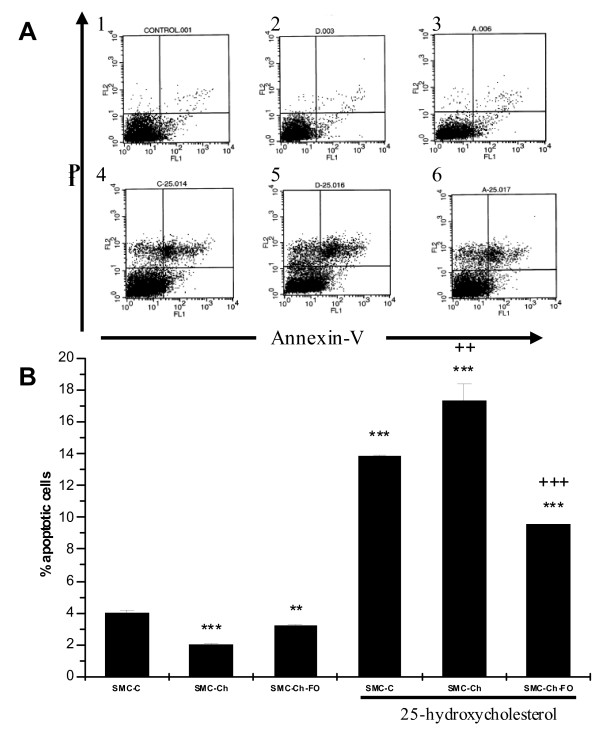
**Apoptosis induced by 25-Hidroxicholesterol in the cell culture model**. Cells were labeled with Annexin V-FITC and PI (propidiun iodide), and apoptosis was assessed by flow cytometry. A, Representative scatter plots of PI (y-axis) versus Annexin V-FITC (x-axis). SMC-C (1), SMC-Ch (2), SMC-Ch-FO (3), SMC-C treated with 20 μg/ml 25-hydroxycholesterol (25-HC) for 24 h (4), SMC-Ch treated with 20 μg/ml 25-HC for 24 h (5), and SMC-Ch-FO treated with 20 μg/ml 25-HC for 24 h (6). Absence of both markers (lower left quadrants) indicates viable cells; PI positive alone (upper left quadrants) indicates cell necrosis, whereas Annexin V staining alone or together with PI (upper right and lower right quadrants) indicates of early- and late-stage apoptosis, respectively. B, Summary data of three experiments showing the % of annexin-V positive cells (upper right and lower right quadrants) in SMC cultures at baseline (SMC-C, SMC-Ch, and SMC-Ch-FO) and after 24-h treatment *in vitro *with 20 μg/ml 25-HC for 24 h. *P < 0.05, ** *P *< 0.01, *** *P *< 0.001 vs. SMC-C, +*P *< 0.05, *++P *< 0.01, +++ *P *< 0.001 vs. SMC-C treated with 20 μg/ml 25-HC for 24 h.

The percentage apoptosis was quantified in the three types of SMC cultures (C, Ch, and Ch-FO) after *in vivo *treatments (baseline) and after *in vitro *treatments. Figure 1 depicts the flow cytometry results and the percentage of annexin V-FITC-positive cells; the percentages in the upper right and lower left quadrants of the Figure represent the SMC in early and late apoptosis at baseline and after 24-h incubation with 20 μg/ml 25-hydroxycholesterol, respectively. At baseline, the SMC showed a very low level of apoptosis (about 4%)

Cells showed a marked increase in apoptosis after the addition of 25-HC (p < 0.001), with a higher increase in SMC-Ch than in SMC-C (p < 0.01).

### Expression of c-myc, p53, and caspase-3 genes in the cell culture model

**Figure 2:**
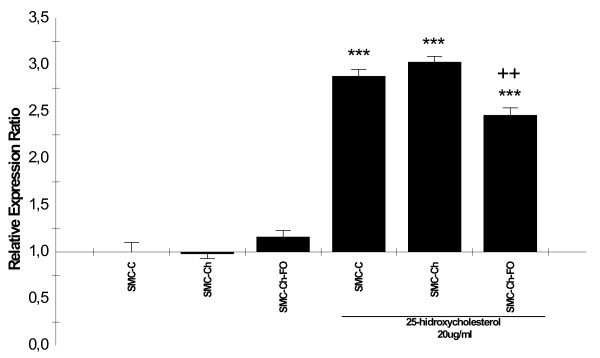
**25-hidroxycholesterol effects on c-myc expression in the cell culture model**. *c-myc *mRNA quantification in SMC cultures (SMC-C, SMC-Ch, SMC-Ch-FO) and SMC cultures treated with 20 μg/ml 25-hydroxycholesterol for 24 h. mRNA levels were quantified by real-time semiquantitative reverse-transcription PCR. Results are shown as relative expression ratio of c-myc in SMC cultures with respect to control culture and expressed in comparison to reference gene β-actin. *P < 0.05, ** *P *< 0.01, *** *P *< 0.001 vs. SMC-C, +P < 0.05, ++P < 0.01, +++ P < 0.001 vs. SMC-C treated with 20 μg/mL 25-hydroxycholesterol.

**Figure 3:**
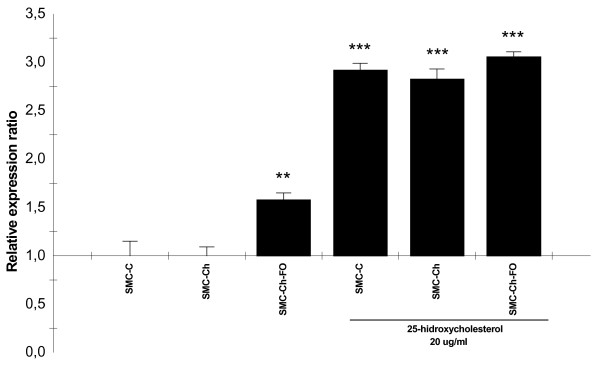
**25-hidroxycholesterol effects on p53 expression in the cell culture model**. *p53 *mRNA quantification in SMC cultures (SMC-C, SMC-Ch, SMC-Ch-FO) and SMC cultures treated with 20 μg/mL 25-hydroxycholesterol for 24 h. mRNA levels were quantified by real-time semiquantitative reverse-transcription PCR. Results are shown as relative expression ratio of p53 in SMC cultures with respect to control culture and expressed in comparison to reference gene β-actin. *P < 0.05, ** *P *< 0.01, *** *P *< 0.001 vs. SMC-C, +P < 0.05, ++P < 0.01, +++ P < 0.001 vs. SMC-C treated with 20 μg/mL 25-hydroxycholesterol.

**Figure 4:**
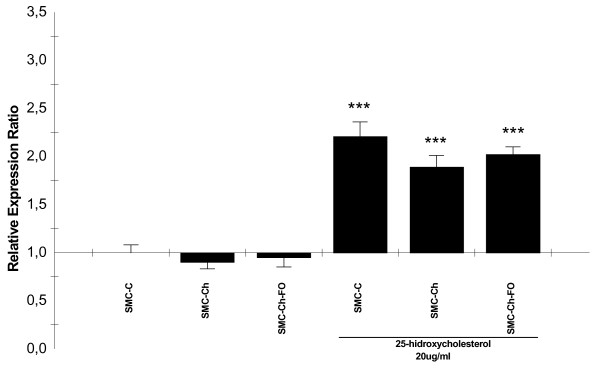
**25-hidroxycholesterol effects on caspase-3 expresión in the cell culture model**. *caspase-3 *mRNA quantification in SMC cultures (SMC-C, SMC-Ch, SMC-Ch-FO) and SMC cultures treated with 20 μg/mL 25-hydroxycholesterol for 24 h. mRNA levels were quantified by semiquantitative real-time reverse-transcription PCR. Results are shown as relative expression ratio of caspase-3 in SMC cultures with respect to control culture and expressed in comparison to reference gene β-actin. *P < 0.05, ** *P *< 0.01, *** *P *< 0.001 vs. SMC-C, +P < 0.05, ++P < 0.01, +++ P < 0.001 vs. SMC-C treated with 20 μg/mL 25-hydroxycholesterol.

At baseline, no significant difference in c-myc expression (Figure 2) was observed among SMC-C, SMC-Ch or SMC-Ch-FO. The expression of p53 gene (Figure 3) was significantly higher in SMC-Ch-FO than in the other two cultures (p < 0.001). None of the SMC cultures showed elevated caspase-3 expression (Figure 4), consistent with the baseline percentages of apoptosis (Figure 1).

### Effect of 25-hydroxycholesterol on the expression of c-myc, p53 and caspase-3 genes

Addition of 25-HC to the cultures produced a significant increase in c-myc expression (p < 0.001), with a significantly lower increase in SMC-Ch-FO versus SMC-C (p < 0.01) (Figure 2). Addition of 25-HC also produced a major increase in p53 mRNA (p < 0.001) in all three culture types (Figure 3). Finally, 25-HC produced a marked increase in caspase-3 gene expression (Figure 4).

## Discussion

The dedifferentiation and proliferation/apoptosis of SMC in the arterial intima are among changes in early atherosclerotic lesions, when the disease is still reversible [37,38]. The very low level of apoptosis shown in SMC in this study is similar to the 4% death rate usually observed in cultured cells. These data are in agreement with previous reports that cultured SMC from the arterial media, even those from atherosclerotic plaque, show no apoptosis in culture, unlike SMC from the intima [39]. In early atherosclerosis, cells in the arterial media that proliferate and migrate, modulating their phenotype, are protected against apoptosis in some manner [40].

SMC from chicks fed for 20 days with a cholesterol-rich diet (SMC-Ch) were more susceptible to apoptosis than those from control chicks on a standard diet (SMC-C). This may be due to the accumulation of lipids in SMC, since lipid accumulation in early atherosclerosis has been associated with cell proliferation [41,42]. In contrast, SMC apoptosis levels were lower in chicks fed for 20 days with fish oil after 20 days on a standard diet (SMC-Ch-FO) than in the control chicks (p < 0.01). This finding can be explained in part by a reversal of the process after the end of the high-cholesterol diet, as observed in animals with experimental atherosclerotic plaques [43], and in part by the protective effect of fish oil against 25-HC-mediated apoptosis, since levels were even lower than in the control SMC.

The expression of p53 gene was elevated in SMC-Ch-FO, supporting the proposition that endogenous levels of p53 protect SMC against apoptosis and possibly against the development of atherosclerosis [44]. The main controversy is whether endogenous p53 predominantly regulates proliferation or apoptosis in SMC. It has been proposed that p53 is responsible for the quiescent state of SMC in the arterial media. During the first changes in early atherosclerosis, SMC acquire the capacity to migrate from the media to the intima, which requires p53 expression inhibition that may be produced by mitogens in the vessels [45], whose signals produce a rapid degradation of endogenous p53 levels. The migration and proliferation of intima SMC commences at this time, and they do not re-express p53 when cultured. If this reduction in endogenous p53 does not occur, the SMC enter into apoptosis.

It has been reported that c-myc expression interferes with the control of SMC proliferation [46], and cultured SMC from atherosclerotic plaque have shown an overexpression of c-myc with respect to control SMC [47]. Elevated LDL levels have been implicated in the increased expression of c-myc in atherogenesis [48]. It should be noted that c-myc is expressed in cells that actively proliferate, and it is able to induce apoptosis in the absence of growth factors [49]. Our results are consistent with findings that oxidized LDLs (oxLDLs) produce an increase in c-myc expression [50]. Hence, dysregulation of c-myc causes SMC to proliferate but simultaneously reduces α-actin and apoptosis levels, corresponding to a change from a contractile to synthetic SMC phenotype [46]. Our observation of lower c-myc levels in SMC-Ch-FO again indicates that these are protected against development of the disease. Oxysterols can produce oxidative stress in cells, thereby activating c-myc gene expression, which is sensitive to oxidative stress processes. Suppression of oxidative stress by the administration of anti-oxidants was found to attenuate this activation and development of the lesion [51]. Oxidative stress is created in areas of lipid accumulation, activating c-myc [51]. This study demonstrated that suppression of oxidative stress by antioxidant administration reduces this activation and progression of the lesion. Our findings indicate that fish oil can reduce the increase in c-myc levels observed in SMC-C and SMC-Ch, possibly through its influence on the expression of antioxidant genes [52].

The significant increase in p53 mRNA in the three culture types is consistent with findings that ox-LDLs (specifically 25-hydroxycholesterol) induce p53 [53]. A marked increase in Bax protein levels was also observed, possibly mediated by p53 [35]. These results indicate that, under our experimental conditions, 25-HC-induced apoptosis is mediated by p53. Finally, the marked increase in caspase-3 gene expression induced by 25-HC is in agreement with reports that oxysterol-induced apoptosis triggers the activation of caspase-3 protein [54].

In **conclusion**, 25-hydroxycholesterol-induced apoptosis of SMC is mediated by a significant increase in c-myc and p53 levels. These changes were generally more marked in SMC-Ch than in SMC-C, indicating that dietary cholesterol produces changes in SMC that make them more susceptible to 25-hydroxycholesterol-mediated apoptosis. Replacement of a cholesterol-rich diet with a fish oil-rich diet produces some reversal of the cholesterol-induced changes, increasing the resistance of SMC to apoptosis.

## Competing interests

The authors declare that they have no competing interests.

## Authors' contributions

All authors participated in the design of these studies and carried out the different assays. A.L. drafted the manuscript. All authors read and approved the final manuscript.
